# Characterization of Protein Hubs by Inferring Interacting Motifs from Protein Interactions

**DOI:** 10.1371/journal.pcbi.0030178

**Published:** 2007-09-14

**Authors:** Ramon Aragues, Andrej Sali, Jaume Bonet, Marc A Marti-Renom, Baldo Oliva

**Affiliations:** 1 Structural Bioinformatics Lab (GRIB), Universitat Pompeu Fabra-IMIM, Barcelona Research Park of Biomedicine (PRBB), Barcelona, Catalonia, Spain; 2 Department of Biopharmaceutical Sciences, University of California San Francisco, San Francisco, California, United States of America; 3 Department of Pharmaceutical Chemistry, University of California San Francisco, San Francisco, California, United States of America; 4 California Institute for Quantitative Biomedical Research, University of California San Francisco, San Francisco, California, United States of America; 5 Structural Genomics Unit, Bioinformatics Department, Centro de Investigación Príncipe Felipe, Valencia, Spain; National Cancer Institute, United States of America, and Tel Aviv University, Israel

## Abstract

The characterization of protein interactions is essential for understanding biological systems. While genome-scale methods are available for identifying interacting proteins, they do not pinpoint the interacting motifs (e.g., a domain, sequence segments, a binding site, or a set of residues). Here, we develop and apply a method for delineating the interacting motifs of hub proteins (i.e., highly connected proteins). The method relies on the observation that proteins with common interaction partners tend to interact with these partners through a common interacting motif. The sole input for the method are binary protein interactions; neither sequence nor structure information is needed. The approach is evaluated by comparing the inferred interacting motifs with domain families defined for 368 proteins in the Structural Classification of Proteins (SCOP). The positive predictive value of the method for detecting proteins with common SCOP families is 75% at sensitivity of 10%. Most of the inferred interacting motifs were significantly associated with sequence patterns, which could be responsible for the common interactions. We find that yeast hubs with multiple interacting motifs are more likely to be essential than hubs with one or two interacting motifs, thus rationalizing the previously observed correlation between essentiality and the number of interacting partners of a protein. We also find that yeast hubs with multiple interacting motifs evolve slower than the average protein, contrary to the hubs with one or two interacting motifs. The proposed method will help us discover unknown interacting motifs and provide biological insights about protein hubs and their roles in interaction networks.

## Author Summary

Recent advances in experimental methods have produced a deluge of protein–protein interactions data. However, these methods do not supply information on which specific protein regions are physically in contact during the interactions. Identifying these regions (interfaces) is fundamental for scientific disciplines that require detailed characterizations of protein interactions. In this work, we present a computational method that identifies groups of proteins with similar interfaces. This is achieved by relying on the observation that proteins with common interaction partners tend to interact through similar interfaces. The proposed method retrieves protein interactions from public data repositories and groups proteins that share a sensible number of interacting partners. Proteins within the same group are then labeled with the same “interacting motif” identifier (iMotif). The evaluation performed using known protein domains and structural binding sites suggests that the method is better suited for proteins with multiple interacting partners (hubs). Using yeast data, we show that the cellular essentiality of a gene better correlates with the number of interacting motifs than with the absolute number of interactions.

## Introduction

Protein–protein interactions play a central role in many cellular processes, ranging from signal transduction to formation of cellular macrostructures and cell cycle control [1–3]. Recently, several techniques such as two-hybrid assays [4–6] and affinity purifications followed by mass spectrometry [7–9] have enabled large-scale identification of protein–protein interactions. While these efforts provide rich lists of interacting proteins, they do not produce information about the specific interfaces involved in each interaction.

Proteins interact through a limited set of interface types [3,10,11]. These interfaces are usually key determinants of the function. Therefore, narrowing down protein–protein interactions to interactions between specific protein components (e.g., a domain, sequence segments, a binding site, or a set of residues) is important for a more accurate characterization of the function of proteins and their complexes. Identifying the protein interfaces that mediate interactions may also be useful for the prediction of unknown protein–protein interactions [12,13], for homology-based protein annotation methods [14], and for relating gene essentiality and network topology [15].

Traditionally, the description of protein interactions in terms of the interacting components has been based on protein structural domains [16], protein functional sites [17], and protein patches [18]. However, fully characterizing protein surfaces that are in contact with each other during an interaction requires the determination of the structure of protein complexes by X-ray crystallography or NMR spectroscopy. These methods are not always applicable and thus the number of known 3-D atomic structures of proteins and their complexes is limited. As a result, accurate and general computational methods for identifying motifs involved in protein–protein interactions are needed.

Recently, several methods [19–25] have been developed to describe protein–protein interactions in terms of interacting protein domains, as defined in the Structural Classification of Proteins (SCOP) [26], PFAM [27], and InterPro [28] databases. However, while these methods find interactions between predefined protein domains, interactions between undefined domains remain undetected. Sequence-based methods overcome this problem by identifying sequence signatures that consistently co-occur in pairs of interacting protein sequences [29], while structure-based methods can predict the amino acid residues that are in contact during a protein–protein interaction, but require information about the structures of both proteins [30–33]. Recently, Kim et al. used known protein interactions and structures to characterize the interfaces between two interacting proteins [15]. They found that some previously accepted relationships between network topology and genomic features [34–36] are actually more reflective of the number of distinct binding interfaces. For example, highly connected proteins in the network (i.e., hubs) with multiple interfaces are twice as likely to be essential as hubs with one or two interfaces. The findings of Kim and coworkers clarify some previous analyses that related the observed essentiality of hubs with their high number of interacting partners [34,37] or with their interactions to other hubs [38]. Kim et al. also demonstrated that the evolutionary rate is significantly lower for multi-interface hubs than for the average protein, but not so for hubs with one or two interfaces.

Here, our basic assumption is that proteins with overlapping sets of interacting partners tend to interact with the common partners through the same interacting motif, such as a domain, sequence segments, a binding site, or a set of residues. A similar assumption has been previously used to annotate protein sequences [14,39–41]. We first tested this assumption based on databases of protein interactions [42] and protein domains defined in SCOP [26], observing that the assumption holds true for highly connected proteins (i.e., hubs in a protein–protein interaction network). Building on this validation, we then developed a method for identifying interacting motifs (iMotifs), which has been implemented within the protein–protein interaction framework and integration engine PIANA (Protein Interactions and Network Analysis) [42]. iMotifs are not required to be of any particular structural type or size, thus allowing us to characterize proteins and their interactions at different levels of resolution, ranging from full proteins to small binding sites. In contrast to other methods, our approach is not limited to finding predefined classes of interacting motifs, such as SCOP domains or PROSITE functional sites, and can be used to identify unknown interacting motifs. Moreover, the sole input for our method is binary protein interactions; neither structure nor sequence information is required to assign iMotifs to proteins.

Two main objectives have been addressed in this work. The first objective was to demonstrate whether protein interactions alone can be used to infer interacting motifs. The positive predictive value of our method in detecting proteins with common SCOP families was 75% at sensitivity of 10%, and the Spearman correlation coefficient between the number of iMotifs assigned to proteins and the number of interfaces found by Kim et al. [15] was 0.57. The second objective was to examine if the conclusions on protein hubs of Kim et al. [15] hold for our iMotifs assignments. The results demonstrate that protein hubs with multiple iMotifs are more likely to be essential than hubs with one or two iMotifs and that protein hubs with multiple iMotifs evolve slower than the average protein in the dataset, as opposed to hubs with one or two iMotifs.

## Results

### Proteins with Common Interaction Partners Tend to Share a SCOP Domain

The basic assumption behind this work is that proteins with overlapping sets of interaction partners tend to interact with those partners through a common interacting motif. The validity of this assumption was tested on a nonredundant set of 368 proteins with known SCOP domains (Material and Methods). Although SCOP does not classify proteins by their interfaces, SCOP domains were used as surrogates for iMotifs because protein interaction types can be defined by the domains in the interacting proteins [43].

We found the number of common interaction partners (*N*) to be a good indicator of the probability of two proteins having a domain in the same SCOP family, especially for highly connected proteins (Figure S1). For example, 73% of protein pairs with 50–60 common interaction partners shared a SCOP domain. We also studied other metrics to measure the similarity between two sets of interaction partners, but none of them outperformed *N* at the identification of protein pairs with a common domain family (Figures S1 and S2A, and Table S1).

It is worth noting that our assumption relies on the binary nature of the input interactions. Two proteins tend to have a common interacting motif only if they share direct physical interactions with the same partner(s). However, the likelihood of two proteins sharing a SCOP domain was lower by solely using yeast two-hybrid experimental data, a detection method that is more likely to contain binary protein interactions than other experimental methods [44] (Figure S2B).

### Delineating Interacting Motifs

**Figure 1:**
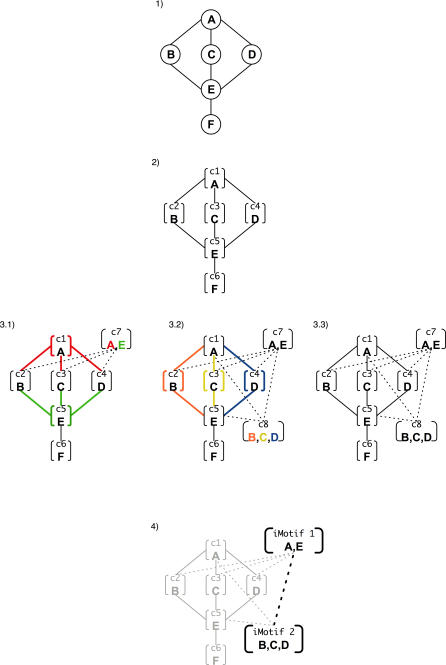
Assigning iMotifs to Proteins and Identifying iMotif–iMotif Interactions First, the protein interaction network is built. Second, a cluster interaction network is created by placing each protein in a different cluster. Third, clustering is performed until the similarity score drops below a certain threshold. Fourth, an iMotif label is assigned to each cluster with more than one protein, and iMotif assignments and interactions are derived.

**Figure 2:**
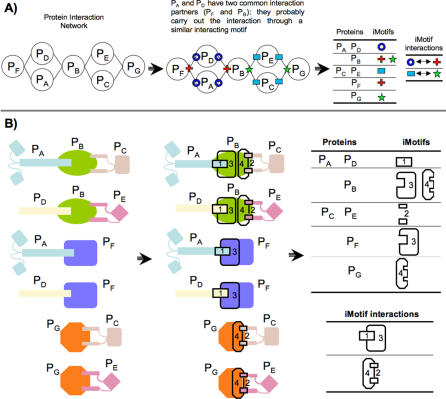
Definition of an Interacting Motif (iMotif) The definition of an iMotif depends on the minimum number of common partners required in order to consider the given binary protein interactions mediated through a common interacting motif. (A) From the protein interaction network perspective, proteins with common partners (two in the example provided) are considered to interact with these partners through a similar feature, and, therefore, are classified as being of the same iMotif. (B) The same process is shown from a structural perspective: proteins interacting through a similar feature (regardless of the feature being two structural domains or a single binding site) are considered to have a common iMotif. To further illustrate the method, we also describe a sample iMotif assignment for *prothrombin* (UniProt code THRB_HUMAN) (Figure S3).

Based on the observation that highly connected proteins with common interaction partners tend to interact with them through a common interacting motif, we have developed a method that groups proteins with similar interacting motifs (Figure 1 and Material and Methods section). Briefly, the procedure is carried out in four steps: 1) build a protein–protein interaction network; 2) initialize a cluster interaction network by assigning each protein of the network to a cluster; 3) iteratively create new clusters by fusing similar clusters (allowing a protein to be in more than one cluster) until the similarity score drops below a predefined threshold; and 4) label with a different interacting motif identifier (iMotif) each cluster with more than one protein and derive iMotif–iMotif interactions from the clustered network. In step 3), the similarity score between two clusters is their number of common interacting partners in the cluster interaction network (*N*). Assigning an iMotif to a group of proteins simply establishes that they have a certain feature that allows them to interact with the same set of partners, without determining the size, sequence, or structure of that feature (Figure 2A). Thus, an iMotif can be an interface consisting of a set of domains or only a specific constellation of a small number of residues (Figure 2B).

### Method Evaluation

The definition of iMotifs depends on a similarity metric and its threshold. Thus, different thresholds or metrics produce different iMotifs, corresponding to different levels of resolution in the description of protein–protein interactions. For example, the method can be applied at the resolution of domains from SCOP [26], and PFAM [27], or at the higher resolution of functional sites from PROSITE [45]. In this section, we have evaluated the method on a nonredundant set of proteins (Material and Methods) for three different tasks: (i) detecting proteins with common SCOP domain families; (ii) predicting SCOP domain–domain interactions observed in the Protein Data Bank (PDB) [46]; and (iii) predicting the number of distinct binding interfaces as defined by Kim et al. [15]. Therefore, in the evaluation, iMotifs effectively represent SCOP family domains (for the first two tasks) and structural binding interfaces (for the third task).

### Detecting Proteins with Domains in the Same SCOP Family

**Figure 3:**
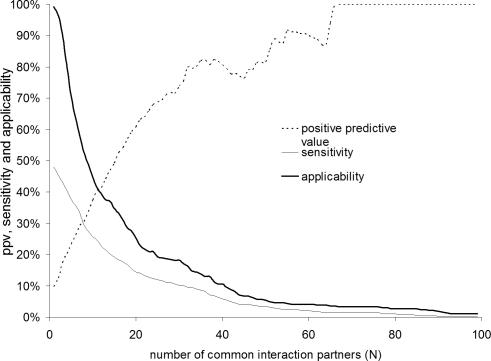
Performance of the Method in Detecting Proteins with Common SCOP Families The positive predictive value, sensitivity, and applicability (Methods) are plotted as a function of the number of common interaction partners threshold (*N*) used for the clustering.

We evaluated the ability of the method to detect proteins with a domain in the same SCOP family (Methods). Using an *N* threshold of 30 common interaction partners, our method achieves a positive predictive value of ∼75%, sensitivity of ∼10%, and applicability of ∼20% (Figure 3). The positive predictive value drops to ∼50% for *N* of 15, indicating that the accuracy of our method in detecting proteins with common SCOP family domains proportionally decreases with the number of common interacting partners. Therefore, the method should be preferentially applied to assigning interacting motifs to highly connected proteins. The growth of the interactome data [47,48] is likely to make the approach more applicable in the future. Nevertheless, the applicability can already be increased at the expense of the positive predictive value by using other similarity metrics (Table S1). We provide a complete list of iMotif assignments for the test set (Table S2).

### Predicting Domain–Domain Interactions

Domain–domain interactions can be predicted from the iMotif–iMotif interactions found by the method (Materials and Methods). We evaluated the accuracy of these predictions with respect to domain interactions in the PDB. Our method achieves a positive predictive value of ∼65% for ∼5% of the proteins in the test set (Figure S4), suggesting that the method can be applied to the prediction of domain–domain interactions when a sufficiently large and varied sample of protein interactions is known. However, with the available interaction data, other methods that rely on both interaction networks and predefined domains [19–22] may be better suited than our approach for predicting domain–domain interactions.

### Predicting the Number of Binding Interfaces

**Figure 4:**
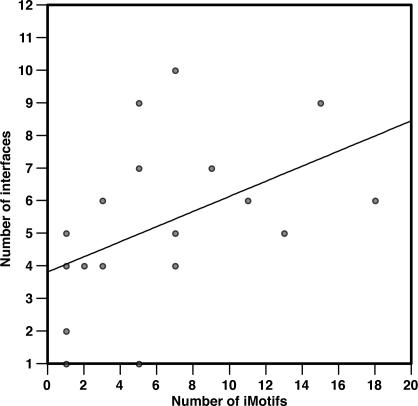
Correlation between the Number of Binding Interfaces and the Number of iMotifs Each point corresponds to a protein from the test set for which a number of binding interfaces was assigned by Kim et al. [15], and a number of iMotifs was inferred with *N* set to 20. Both variables were found to be significantly correlated (*r_s_* is 0.57 and *p*-value is 0.01). The correlation between the number of interfaces and the number of iMotifs is significant for all *N* values lower than 23 (Figure S5).

Kim et al. used protein 3-D structures and binary protein interactions to make inferences about the number of binding interfaces of proteins [15]. We tested whether there is a correlation between the number of binding interfaces found in their work and the number of iMotifs predicted by our method (Figure 4). The number of protein interfaces indeed correlates with the number of predicted iMotifs per protein (e.g., for *N* of 20, *r_s_* is 0.57 and *p*-value 0.01). The number of iMotifs assigned to proteins by our method tends to be higher than the number of binding sites defined by Kim et al. This might be attributed to two factors: (i) current structural data do not contain all possible protein–protein interactions, resulting in an underestimation of the number of binding sites assigned by the method in [15], and (ii) the lack of coverage of the interactome space, which results in an overestimation of the number of iMotifs per protein assigned by our method. The second factor is addressed by using sequence information to merge similar iMotifs (below).

### iMotifs Assignments for Hub Proteins

Using an *N* threshold of 20, our method assigned 12,342 iMotifs to 2,014 of the 5,571 hub proteins in PIANA (i.e., proteins with 20 or more interaction partners), resulting on average in 8.6 iMotifs per hub. The percentage of hubs with one or two iMotifs was 46% (241 hubs had one iMotif; 689 hubs had two iMotifs). We studied the correlation between the number of iMotifs assigned to a hub and its number of interactions, finding no relationship between the two variables (Spearman correlation coefficient is −0.002 with *p*-value 0.94). A complete list of iMotif assignments for all hub proteins in PIANA is in Table S3 and the number of iMotifs per hub is in Table S4.

### Essentiality and Number of iMotifs Are Correlated in Hub Proteins

**Figure 5:**
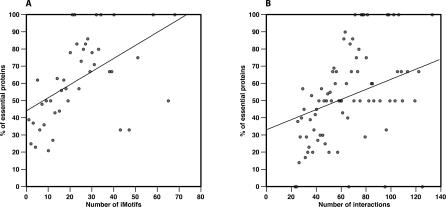
Correlation between the Number of iMotifs and Protein Essentiality Proteins from PIANA were binned according to their number of iMotifs (A) and to their number of interactions (B), and the fraction of essential proteins was calculated for each bin. Bins with only one protein were not considered for calculating the correlations. (A) Correlation between the number of iMotifs assigned to yeast hub proteins (≥20 interactions) in PIANA and the fraction of essential proteins (*r_s_* is 0.61 and *p*-value is 1.6 × 10^−5^). iMotifs were assigned to yeast hubs using an *N* threshold of 20. (B) Correlation between the number of interactions of yeast hub proteins in Figure 5A and the fraction of essential proteins (*r_s_* is 0.51 and *p*-value is 1.1 × 10^−6^).

Similarly to Kim and co-workers' results, [15], we found that yeast hubs with multiple iMotifs are more likely to be essential than those with one or two iMotifs (singlish-iMotif) (Table 1). Furthermore, we observed a correlation (*r_s_* is 0.61 and *p*-value is 1.64 × 10^−5^) between the number of iMotifs in yeast hubs and the fraction of essential proteins (Figure 5A). We compared the correlation between iMotifs and essentiality to the correlation between the number of interactions of hubs and essentiality to confirm that the first was not a direct consequence of the second (Figure 5B). These results suggest that the number of iMotifs predicted for a protein could be used for selecting biologically relevant candidates for gene deletion experiments.

**Table 1:**
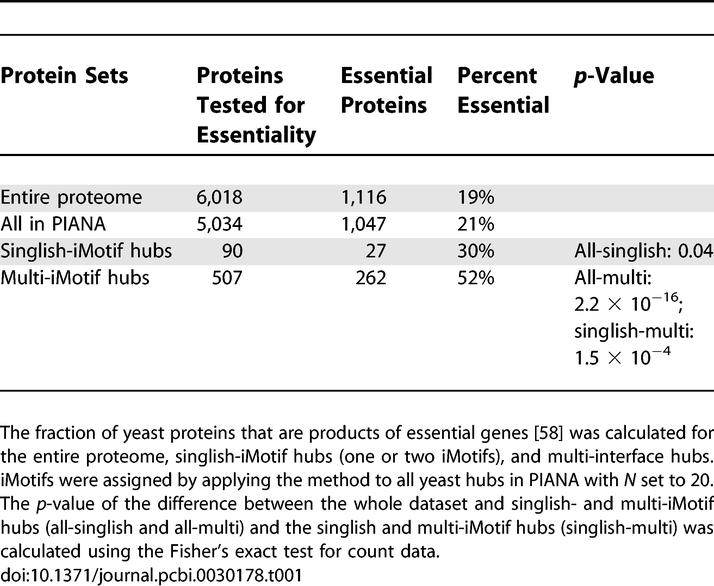
Protein Essentiality and Predicted iMotifs

### Multi-iMotif Hubs Evolve Slower Than Other Proteins; Singlish-iMotif Hubs Do Not

A common measure of evolutionary rate is the dN/dS ratio (the ratio of nonsynonymous to synonymous substitutions) [49]. Kim et al. found that multi-interface hubs have a lower evolutionary rate than the average protein in their data, but the same was not true for singlish-interface hubs. Our results are in agreement with their findings. Multi-iMotif hubs, in contrast to singlish-iMotif, evolve significantly slower than the average protein in our dataset (Table 2). However, the evolutionary rate difference between multi- and singlish-iMotif hubs (i.e., 0.062 and 0.056, respectively) was not found to be significant (*p*-value of 0.21).

**Table 2:**
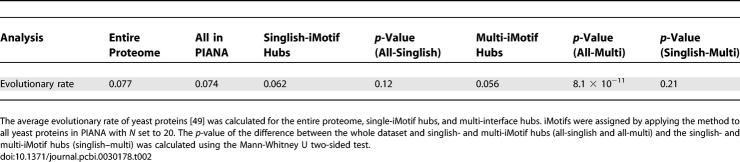
Protein Evolutionary Rate and Predicted iMotifs

### Extracting Sequence Patterns from iMotifs

**Figure 6:**
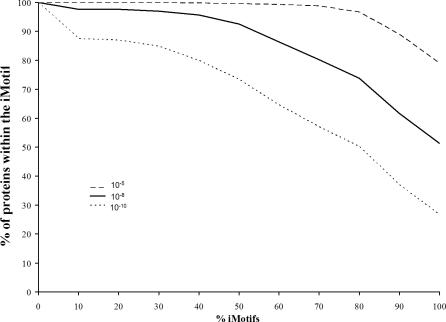
Sequence Patterns in iMotifs Relationship between the percentage of iMotifs for which a significant sequence pattern was found and the percentage of proteins within the iMotif that contained the pattern. Three different significance cutoffs were used for associating sequence patterns to iMotifs: 10^−5^ (long dashed line), 10^−8^ (solid line), and 10^−10^ (short dashed line).

Sequence patterns for each iMotif were generated using the PRATT program [50] (Methods). Briefly, PRATT identifies sequence patterns common to a set of sequences. In this work, we selected significant sequence patterns for each iMotif by maximizing the number of proteins within the iMotif that contained the pattern. The significance (i.e., *p*-value) of a sequence pattern assigned to an iMotif depends on the occurrence of the pattern in the iMotif with respect the whole dataset. As shown on Figure 6, 80% of iMotifs had a specific sequence pattern contained in at least 74% of their proteins (using a *p*-value cutoff of 10^−8^). A list with the best sequence pattern for each iMotif is provided in Table S5. Interestingly, a similar analysis based on Pfams assignments to iMotifs showed a different trend (i.e., very few iMotifs had most of their proteins described by a Pfam). For example, as shown on Figure S6, only 10% of all iMotifs had a specific Pfam in at least 28% of their proteins (*p*-value cutoff of 10^−8^). Such a difference can be explained by the fact that many interactions are carried out by short sequence patches [3,51], while Pfam families usually consist of long structured protein regions.

As indicated above, incompleteness in interaction data may result in artificially high numbers of iMotifs. This overestimation can be reduced by merging iMotifs with a common sequence pattern (Material and Methods). Fusing iMotifs based on sequence pattern similarity decreased the average number of iMotifs per hub from 8.6 to 4.2. This reduction, in turn, increased the correlation between the number of binding sites from Kim et al. [15] and the number of iMotifs in the test set proteins (Spearman correlation coefficient was 0.59 with *p*-value of 0.001).

## Discussion

We described, implemented, and evaluated a method that relies solely on binary protein interactions to identify interacting motifs (iMotifs) and their interactions. Our approach obtained high positive predictive value for identifying proteins with domains from the same SCOP family and predicting domain–domain interactions. We also analyzed hub proteins and their properties based on the number of iMotif assigned to them, obtaining similar findings to those in an independent approach that rely on protein structure information [15].

Recent estimates suggested that only one-fifth of interaction types are known [43]. Therefore, current knowledge of protein structures is not sufficient to describe all protein interaction types. Our approach, in contrast to other previously described methods, accomplishes three different objectives: (i) it predicts the number of different iMotifs in a protein, (ii) it classifies proteins by their predicted iMotifs, and (iii) it predicts interactions between the iMotifs. The method can identify iMotifs independently of structural or sequence information; it can assign an iMotif to two structural domains or two iMotifs to a single domain. Since the resolution at which iMotifs describe protein interfaces depends on the similarity metric used and the threshold applied by the method, iMotif assignments can be used to infer whether the interaction is mediated through multiple, single, or partial domains. On the one hand, setting a high threshold on the number of common interaction partners (*N*) will assign few iMotifs to reduced sets of proteins (i.e., very specific and restrictive iMotifs). On the other hand, using low *N* thresholds will assign the same iMotif to broad numbers of proteins (i.e., very unspecific and general iMotifs). We showed that the method works better for highly connected proteins and using high values for *N*. Moreover, our approach is not limited to finding predefined classes of protein components and thus allows us to predict new types of interacting motifs. For example, an iMotif can be mapped to a predefined class (e.g., a SCOP domain or a PROSITE functional site) by examining the known classes assigned to proteins with that iMotif. Therefore, iMotifs that remain unmapped are likely candidates for unknown classes. Such predictions may prove useful for target selection in structural genomics.

Relying solely on experimentally detected interactions affects the accuracy of our method. It has been shown that high-throughput experiments have limited reliability and that many of the detected interactions are probably not direct (i.e., they are carried out through a third protein) or do not even exist (i.e., false positives) [52]. However, we did not observe an improvement by solely using interactions from yeast two-hybrid assays (Figure S2B), the high-throughput method that is best suited to detect direct interactions. As more interaction data becomes available, we will reexamine the effects of restricting the method to employ interactions specifically labeled as “direct” [6]. One way of avoiding these limitations is to calculate similarity scores using families of proteins instead of absolute numbers of protein partners. This will prevent assigning the same iMotif to proteins that have many common partners but all of them belong to a single protein family. Removal of redundancy from the sets of partners indeed increases the percentage of identified protein pairs with a common domain family (Figure S7).

The combination of iMotif assignments with sequence search methods identified specific sequence patterns in iMotifs. We found that most iMotifs had a significant sequence pattern that was contained in most of the iMotif proteins. These patterns, which could be responsible for the iMotif proteins common interactions, could then be used to: (i) localize the iMotif in the protein sequence, (ii) assign iMotifs to proteins for which no interaction data is yet available, and (iii) predict interactions between proteins that contain patterns assigned to two interacting iMotifs.

Our iMotif assignments are similar to those obtained using an independent approach, which relies not only on known protein–protein interactions, but also on protein structure information [15]. In agreement with the results of Kim et al.*,* we observe different properties between hubs with multiple iMotifs (multi-iMotif) and hubs with one or two iMotifs (singlish-iMotif). In particular, we find that (i) multi-iMotif hubs are more likely than singlish-iMotif hubs to be essential for cell viability, and (ii) multi-iMotif hubs, in contrast to singlish-iMotif hubs, evolve slower than the average protein. Furthermore, we have also observed a correlation between the number of iMotifs of a hub and its essentiality for cell survival. The properties observed for hubs with respect to their number of iMotifs may reflect the difference between proteins with multiple simultaneously possible interactions (multi-iMotif hubs are probably involved in permanent complexes) and proteins with multiple exclusive interactions (for singlish-iMotif hubs involved in transient interactions). This is in agreement with the previous observation that interfaces of transient protein–protein interactions are less restricted in evolution than interfaces in permanent complexes [53].

Our results extend the findings and conclusions of Kim and co-workers [15] to proteins of unknown structure. Thus, inferring interacting motifs from protein interactions is likely to be helpful for providing biological insights about hubs for which no structural information is available.

## Materials and Methods

### Protein interactions.

Protein–protein interactions from DIP 2006.01.16 [54], MIPS 2006.01 [55], HPRD 2005.09.13 [56], BIND 2006.01 [57], and two recent high-throughput experiments [5,6] were integrated using PIANA version 1.2 [42], allowing us to work with a large set of 363,571 interactions between 42,040 proteins. PIANA represents protein interactions as a network where the nodes are proteins and the edges are interactions between the proteins. In such a network, a set of proteins linked to protein *p_j_* (i.e., physically interacting with *p_j_*) is named “partners of *p_j_*”. In such a network, we define hubs as proteins with 20 or more partners. The average number of interactions per hub in our dataset was ∼49. PIANA builds the protein interaction network by retrieving partners for an initial set of proteins. To avoid a positive bias in the method evaluation, interactions inferred from 3-D structures were not used in this work.

### Structural domains and protein binding interfaces.

Protein domain assignments and classification were obtained from the SCOP release 1.69 [26]. Here, domains are defined at the SCOP family level. Thus, domain–domain interactions refer to SCOP family interactions. The number of protein binding interfaces for hub proteins was obtained from the Structural Interaction Network 2.0 [15].

### Essential proteins and evolutionary rates.

A list of ORFs essential for the survival of the yeast cell was obtained from the *Saccharomyces* Genome Deletion Project [58]. The evolutionary rates (dN/dS) of yeast proteins were taken from the adjusted values given by Wall et al. [49].

### Assigning iMotifs to proteins and finding iMotif–iMotif interactions.

The assignment of iMotifs to a set of proteins is carried out in a four-step procedure (Figure 1):

First, build the protein interaction network.

Second, initialize a cluster interaction network (i.e., nodes are clusters that contain one or more proteins, and edges are interactions between clusters) by assigning each protein of the protein interaction network to a different cluster. Each cluster (containing one protein *p_j_*) interacts with those clusters that contain a partner of *p_j_* in the protein interaction network.

Third, iteratively create new clusters by fusing the most similar clusters until the similarity score drops below a predefined threshold. Two clusters are similar if they share a minimum number of common interacting partners (*N*). Thus, the similarity score between two clusters is their number of common partners in the cluster interaction network. Other similarity metrics were considered, but none outperformed the use of *N* (Figure S1). When fusing two clusters, the resulting cluster inherits interactions that were common to both fused clusters. Since proteins may have multiple interfaces, all initial clusters (from step 2) remain in the cluster interaction network even after being fused to another cluster. Interactions between non-initial clusters are not considered for calculating the similarity scores.

Fourth, each cluster with more than one protein is labeled with a different interacting motif identifier (iMotif), and that iMotif is assigned to all proteins within that cluster. iMotif–iMotif interactions are then derived from interactions in the cluster interaction network where both sides of the interaction have been labeled with an iMotif identifier.

For example (Figure 1), a proteome of six proteins (namely A, B, C, D, E, and F) forms a network of interactions that connects proteins A with B, C, and D, and protein E with B, C, D, and F (step 1). Our method starts by creating a cluster interaction network from the network of protein interactions (i.e., six clusters with seven interactions) (step 2). Next, the clusters that share the largest number of common interactions are fused (i.e., clusters 1 and 5, with three common interactions, are fused into a new cluster 7). This step is then repeated until the maximum similarity score between the clusters drops below a predefined threshold (i.e., *N* = 2 common interactions). Thus, the iterative process will run for another iteration creating a new cluster (cluster 8) by fusing clusters 2, 3, and 4, which have two common interactions (step 3). Once the iterative process is finished, the method assigns iMotif identifiers to all proteins in clusters with more than one protein (i.e., proteins A and E in cluster 7 share iMotif 1, and proteins B, C, and D in cluster 8 share iMotif 2) (step 4). Moreover, iMotif–iMotif interactions are then derived from the cluster interaction network (i.e., one interaction between iMotif 1 and iMotif 2).

Figure 2 illustrates iMotif assignments from a network perspective (Figure 2A) and from a structural perspective (Figure 2B). A more detailed description of the algorithm is provided as pseudocode in Figure S8.

### Test set and evaluation procedure.

We have evaluated the method on a test set created by selecting proteins (i) with at least five experimentally detected interactions, (ii) with at least 80% of their sequence covered by the domains defined in SCOP, and (iii) that did not introduce a redundancy bias in the evaluation (i.e., if any two sequences had a sequence identity greater than 30%, a BLAST e-value smaller than 10^−5^, and the alignment had at least 30 residues, the shortest member of the pair was not selected). The final set contained 368 sequences (Table S6). Due to the restrictions imposed, the test set contains many proteins related to the proteosome and the ribosome.

The SCOP family assignment was evaluated by considering as positive assignments those proteins found by the method to have a common iMotif with the query protein. Among these positives, we define as true positives those proteins that have a common SCOP family code with the query protein. Moreover, we define as false negatives the proteins that have the same SCOP family code as the query protein but were not found by the method to share an iMotif.

iMotif–iMotif interaction predictions were evaluated against interacting SCOP families obtained from the PDB. Two SCOP domains were considered to interact if they were co-crystallized and had at least two atoms within 5 Å distance. Because we are interested in domain interactions at the protein–protein interaction level, we excluded intrachain interactions from this set. Our method creates a list of putative domain–domain interactions for each predicted iMotif–iMotif interaction by assuming that all domains of the query protein with one iMotif interact with all domains of proteins with the other iMotif. In this context, we define as positive any iMotif–iMotif interaction where the query protein is involved. A positive is then considered a true prediction if at least one of its putative domain–domain interactions is observed in the PDB. Finally, false negatives are interactions observed in the PDB for SCOP families of the query protein that do not appear in any list of putative SCOP family interactions.

To avoid biases in the evaluation, only proteins from the test set (before removing redundancy) and their SCOP families were considered when counting positives and negatives. The positive predictive value is defined as the number of true positives over the total number of positives, and sensitivity is the number of true positives over the sum of true positives and false negatives. The positive predictive value and sensitivity were calculated with respect to the similarity score threshold used for stopping the clustering. We also define the applicability of the method as the percentage of proteins with at least one positive under a given threshold.

### Extracting sequence patterns from iMotifs.

For each group of protein sequences with a given iMotif, sequence signatures were generated using the PRATT program [50], a software tool capable of finding flexible sequence patterns from a set of unaligned sequences. Parameters were set to produce patterns covering a maximum of 15 residues with no more than three consecutive unspecified positions (gaps) and a maximum of one flexible (of variable length) gap region. The number of nonredundant patterns for all iMotifs was 80,654. Next, all patterns were searched against all proteins in PIANA with at least one interaction (a dataset of 42,040 sequences) using the ps_scan program [59].

The significance (i.e., *p*-value) of the association between a sequence pattern and an iMotif was assessed using the binomial distribution, based on the occurrence of the pattern inside the iMotif with respect the whole dataset [60]:

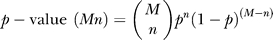
where *M* is the number of protein sequences within an iMotif, *n* represents the number of proteins within the iMotif that contain the sequence pattern, and *p* is the probability of finding a protein from the whole dataset that contains the same pattern (i.e., the number of proteins containing the pattern divided by the number of proteins in the whole dataset).

### Best iMotif sequence pattern.

In this work, the best sequence pattern for each iMotif (Table S5) was considered to be the pattern that maximized the number of proteins in the iMotif that had the pattern. Two additional considerations were taken into account for selecting the best pattern for an iMotif: (i) the sequence pattern should be found in at least 70% of proteins within the iMotif and (ii) the *p*-value of the pattern should be lower than 10^−8^.

### Merging iMotifs based on sequence commonalities.

Interacting motifs were merged by means of an agglomerative hierarchical clustering. Two iMotifs were considered to be similar if they had a common sequence pattern when applying a *p*-value cutoff of 1 × 10^−5^ and requiring the pattern to be found in at least 70% of proteins in both iMotifs. Using more stringent *p*-value cutoffs did not produce any iMotif fusions for proteins from the test set.

### Assigning Pfams to iMotifs.

Hidden Markov Models from the Pfam-A database [61] were assigned to all proteins with at least one known interaction (a dataset of 42,040 sequences) using the HMMER package [62]. The *p*-value for each HMM in relation with each iMotif was calculated using the binomial distribution, based on the occurrence of the Pfam inside the iMotif and in the whole dataset (above).

### Statistical tests.

All correlations were measured using the Spearman rank correlation coefficient (*r_s_*). The assessment of whether two binomial samples of essentiality observations are significantly different was calculated using Fisher's test. The assessment of whether two non-Gaussian samples of evolutionary rate observations come from the same distribution was calculated using the Mann-Whitney U two-sided test. Correlations and differences in the observations were considered significant for *p*-values lower than 0.05. All tests were performed using the implementation provided by *R* [63].

## Supporting Information

Figure S1 The Percentage of Protein Pairs Having a Domain of the Same SCOP Family Is Plotted as a Function of Their Similarity Scores (Grouped in Ranges of 10 Units) To measure the likelihood of two proteins *p_i_* and *p_j_* having a common interacting motif, we defined four different similarity metrics: 1) *N*: the number of interaction partners that are common to *p_i_* and *p_j_* (long dashed line); 2) *R_max_*: the ratio between *N* and the number of partners of the protein with more partners (bold line); 3) *R_min_*: the ratio between *N* and the number of partners of the protein with fewer partners (circles); 4) *R_ave_*: the average of metrics *R_max_* and *R_min_* (dotted line). For each score obtained using the similarity metrics described above, the percentage of protein pairs within that score range is plotted. For example, we observed that using *N* as the similarity metric, 73% of proteins with 50–60 common interaction partners shared a SCOP domain.
(94 KB TIF)

Figure S2 The Percentage of Protein Pairs Having a Domain of the Same SCOP Family Is Plotted as a Function of Their Similarity Scores (Grouped in Ranges of 10 Units), Using the Same Parameters as in Figure S1 but Introducing New Restrictions (A) Proteins that have more than 70 interactions are ignored when performing the analysis.
(B) Only interactions from y2h are used.
(94 KB TIF)

Figure S3 Sample iMotif Assignment (A) Superposition of the prothrombin and the pancreatic trypsin inhibitor structures (PDB IDs 1BTH and 2HPQ) shows an interaction through the SCOP family domain Eukaryotic proteases (in red).
(B) The structure of the anionic trypsin II interaction with the pancreatic trypsin inhibitor (PDB ID 1BRB) also shows an interaction through the SCOP family domain Eukaryotic proteases (in red).
(233 KB TIF)

Figure S4 Performance of the Method in Predicting SCOP Domain–Domain Interactions The positive predictive value, sensitivity, and applicability are plotted as a function of the number of common interacting partners threshold used for the clustering. The positive predictive value and sensitivity using a trivial approach are also shown (thin lines). The applicability for the trivial approach is ∼70%. Sensitivity is highly dependent on the group of proteins for which an iMotif–iMotif interaction can be predicted at a given threshold: if a protein with a prediction has a SCOP code with multiple interactions in the PDB, the sensitivity obtained can vary greatly from one threshold to another. Moreover, we compared our method with the trivial approach of creating putative lists of domain–domain interactions by assuming that all domain families of proteins in the test set interact with all domain families of their interaction partners. The positive predictive value for this trivial approach was 33%, which is below that of our method for thresholds higher than 15.
(93 KB TIF)

Figure S5 Spearman Correlation Coefficient between the Number of Interfaces and the Number of iMotifs Is Plotted as a Function of Different *N* Thresholds Due to the limited number of iMotif assignments with stringent N thresholds, correlations become nonsignificant (i.e., *p*-value > 0.05) for N thresholds higher than 22.
(35 KB TIF)

Figure S6 Relationship between the Percentage of iMotifs for Which a Significant Pfam Was Detected by Sequence Search and the Percentage of Proteins within the iMotif That Contained the Pfam Three different significance cutoffs were used for finding Pfams associated with iMotifs: 10–5 (long dashed line), 10–8 (plain line), and 10–10 (short dashed line).
(87 KB TIF)

Figure S7 The Percentage of Protein Pairs Having a Domain of the Same SCOP Family Is Plotted as a Function of Their Similarity Scores (Grouped in Ranges of 10 Units), Using the Same Parameters as in Figure S1 but Introducing a New Restriction: Redundancy Was Removed from the Sets of Partners To Avoid Artificial Increase or Decrease of the Score Caused by Groups of Homolog Proteins The procedure followed to remove redundancy was the same as the one used for creating the evaluation set. We observe a significant improvement for all metrics with respect to Figure S1.
(95 KB TIF)

Figure S8 Pseudocode of the Algorithm Implemented for Assigning iMotifs to Proteins from a Protein–Protein Interaction Network (345 KB TIF)

Table S1 Number of Protein Pairs under Each Similarity Score Range for Metrics Described in Figure S1
In parentheses, the number of pairs with at least one domain within the same SCOP family is indicated. We observe that metrics such as Rmin outperform N at detecting a higher number of protein pairs with a domain within the same SCOP family, but this is done at the expense of decreasing the accuracy of the method.
(63 KB PDF)

Table S2 Complete List of iMotifs Assignments for Proteins in the Test Set The number of common interaction partners (N) was set to 15. First column is the iMotif identifier. Second column is the number of proteins within the iMotif. Subsequent colums are the proteins within the iMotif. Proteins are identified using UniProt entry names and NCBI GI identifiers. In parentheses, “yes”, “no”, and “-” indicate whether the proteins had a domain within the same SCOP family.
(251 KB TXT)

Table S3 Complete List of iMotifs Assignments for Hub Proteins in PIANA The number of common interaction partners (N) was set to 20. See legend on Table S2.
(2.4 MB TXT)

Table S4 Number of iMotifs Assigned to Each Hub in PIANA (38 KB TXT)

Table S5 Best Sequence Pattern for Each iMotif from Table S3
First column is the iMotif identifier. Second column is the pattern in PROSITE format. Third column is the fraction of proteins within the iMotif that have the pattern. Fourth column is the *p*-value of the pattern.
(704 KB TXT)

Table S6 Click here for additional data file. Proteins from the Test Set, using UniProt Accession Numbers
(68 KB PDF)

## Abbreviations

iMotif: interacting motif
PDB: Protein Data Bank
PIANA: Protein Interactions and Network Analysis
SCOP: Structural Classification of Proteins
