# Supplementary material for: Characterization of Protein Hubs by Inferring Interacting Motifs from Protein Interactions
Source: PLoS Comput Biol. 2007 Sep 14;3(9):e178. doi: 10.1371/journal.pcbi.0030178 (PMC1976338; doi:10.1371/journal.pcbi.0030178)

SI Figure S3: example of iMotif assignment

Sample iMotif assignment. A) Superposition of the prothrombin and the pancreatic trypsin inhibitor structures (PDB ids 1BTH and 2HPQ) shows an interaction through the SCOP family domain Eukaryotic proteases (in red). B) The structure of the anionic trypsin II interaction with the pancreatic trypsin inhibitor (PDB id 1BRB) also shows an interaction through the SCOP family domain Eukaryotic proteases (in red).

Prothrombin is known to interact with the *pancreatic trypsin inhibitor* (BPT1_BOVIN), which is in turn a known interaction partner of the *anionic trypsin II* (TRY2_RAT). Therefore, our method determines that (i) *prothrombin* has two iMotifs (because it has other interactions not shared with the trypsin); (ii) *prothrombin* and *anionic trypsin II* have a common iMotif; and (iii) the common iMotif is responsible for the interaction with the *pancreatic trypsin inhibitor*.

Prothrombin is involved in blood coagulation and consists of a Gamma-carboxyglutamic acid-rich (Gla) domain, two Kringle domains, and one Serine-protease domain of the trypsin family. The Serine-protease domain is responsible for the cleavage of peptide bonds, the Gla domain binds to the membrane, and the kringle domains are involved in the regulation of the proteolytic activity. The enzymatic activity of *prothrombin* is similar to that of *trypsin* (the catalytic domain is in the same SCOP family as those of trypsin-like proteases). A comparison of the interactions *trypsin inhibitor* (BPTI) – rat *trypsin* (PDB id 1BRB) and BPTI -- *prothrombin* (PDB ids 1BTH and 2HPQ) shows that the iMotif identified by our method corresponds to the common catalytic domain. This example illustrates how a common interacting motif can be identified without recourse to comparing sequences or structures.

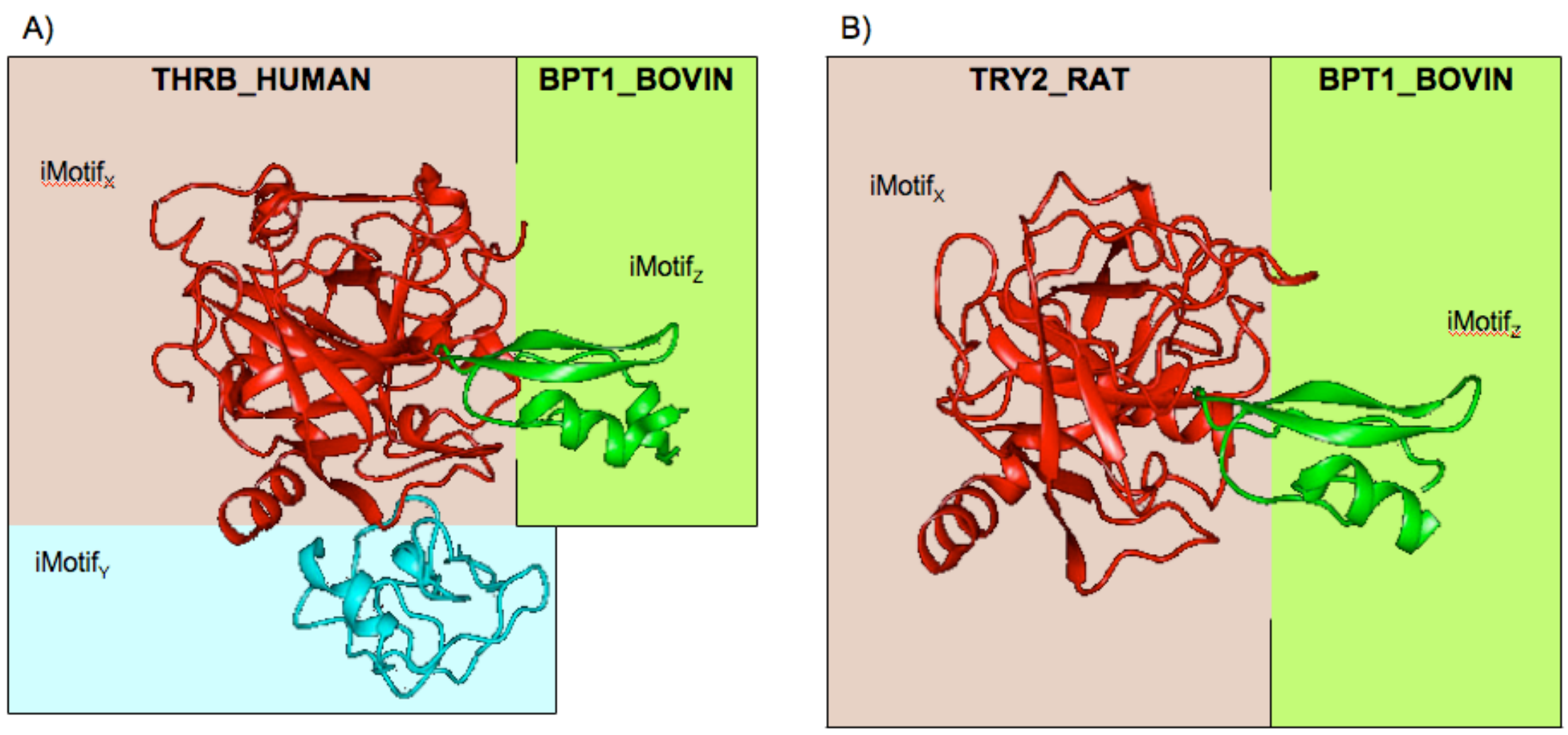

Supplement: Supplementary file 1 [file pcbi.0030178.sg003.pdf]
