# Supplementary material for: Characterization of Protein Hubs by Inferring Interacting Motifs from Protein Interactions
Source: PLoS Comput Biol. 2007 Sep 14;3(9):e178. doi: 10.1371/journal.pcbi.0030178 (PMC1976338; doi:10.1371/journal.pcbi.0030178)
Supplement: Supplementary file 2 [file pcbi.0030178.st001.pdf]

Table S1. Number of protein pairs under each similarity score range for metrics described in Fig. S1. In parenthesis, the number of pairs with at least one domain within the same SCOP family is indicated. We observe that metrics such as R_{min} outperform *N* at detecting a higher number of protein pairs with a domain within the same SCOP family, but this is done at the expense of being less precise.

Similarity Score range	R_{ave}	R_{max}	R_{min}	N
0	62296 (243)	62296 (243)	62296 (243)	62296 (243)
01-10	1678 (26)	3271 (92)	995 (21)	4487 (122)
11-20	1719 (41)	1053 (73)	1446 (22)	352 (66)
21-30	904 (60)	371 (49)	938 (39)	68 (22)
31-40	407 (53)	108 (21)	812 (37)	30 (17)
41-50	182 (55)	39 (13)	344 (39)	26 (21)
51-60	63 (17)	17 (7)	175 (26)	11 (8)
61-70	12 (3)	9 (3)	130 (36)	6 (5)
71-80	9 (4)	6 (5)	86 (26)	3 (3)
81-90	11 (9)	5 (4)	36 (17)	5 (5)
91-100	4 (2)	3 (1)	27 (7)	1 (1)
