# Supplementary material for: Characterization of Protein Hubs by Inferring Interacting Motifs from Protein Interactions
Source: PLoS Comput Biol. 2007 Sep 14;3(9):e178. doi: 10.1371/journal.pcbi.0030178 (PMC1976338; doi:10.1371/journal.pcbi.0030178)
Supplement: Supplementary file 3 [file pcbi.0030178.st006.pdf]

Table S6. Proteins from the test set, using UniProt accession numbers

Uniprot accession number
P62877
P0AF03
P0A7V0
P0A6P1
Q15819
P0A7S3
P37351
P62483
Q02790
P0A6L9
P0A7W1
P09327
O60880
P14900
P24183
P09373
P0A6M4
P02741
P0ACJ8
Q15369
Q14974
P07257
P00492
P0A6F9
P12004
P42224
P10415
O15145
P0A7J0
P27694
P07738
P04802
P09030
P27487
P04995
Q13564
P59998
P30749
P69797
P43577
P02930
P32377
Q5TZP7
P35609
Q05871
P0A6C1
P02932
P60953
P00452
Q99828
Q53RU0
P16083
P67910
P26783
P07170
P0AC81
P0A6W5
P15034
P22139
P14174

P54999
P18754
P0A7G6
Q45QG9
P63165
Q32ML5
P41807
P05112
P02787
Q00403
O43924
P32324
P76015
P75960
P14120
P0C0R7
P28631
P25043
P28630
P63096
P11880
P0ABS8
P63177
P30626
Q12341
P62826
P62988
P00962
P22188
P32774
P0A738
P0AAI5
O14717
P68919
P33552
P22301
P99999
P0A8Y5
P0AFG6
P0A749
P00582
P15309
P30748
P08107
P0A6E6
P06169
P20436
P45748
P29373
P68036
P06738
P0A6U5
P62399
P0A7W7
P0ABH9
P68699
Q06609
P0A8A0
P0A7R9
P0A7V8
P02774
P20606
P50465
P06612

Q99418
P31663
P0A780
Q6FI08
P60723
P0A7J7
P55957
P49841
P0A6A8
P49366
Q06592
P69924
Q6IBS9
P52298
P0AGK4
P61949
P39958
Q14376
P0A720
P0A7B8
P23528
P27999
P0AG30
P60484
Q3SWW1
P0AEK4
P52286
Q06323
P04179
P09624
P37837
P06730
Q6FHR2
Q06549
Q08209
P26602
Q13616
P60520
P0A722
P02994
P38902
P09467
P17443
P56279
P0A8M3
P08133
P0AA43
P13489
P61769
Q32NC1
P26447
P16622
P05120
P0A7R5
P0A6B7
Q6FGE3
P0A8F8
P0A9Z1
P54885
P0A9X9
P0A6N1
Q96CW1
P0A6Q3
P26441

P0ABQ4
P03956
P10619
P00488
P0ACC7
Q6FHM6
P61970
P42773
P11413
Q5D0F4
P34896
P0A715
P32125
P08519
P21242
P69488
P00918
P0AC38
P0A8N3
P29468
P09382
P48736
P0A7S9
P38074
P32939
P32173
P0AB83
P07858
P23284
P24182
P05748
P63212
P49354
P01033
O00762
P0A7L0
P0A6I6
P00491
P22141
P55211
P05121
P0A6F1
P51946
P09938
P53141
P49137
Q3KP48
P0AGE9
P0A6H5
P67870
P0A988
Q53Y44
P0A6R3
P0A7T3
P63010
P18510
P0A8H8
P11177
Q13257
Q71DJ3
P11914
P30657
P78417
P00924

P12281
P0A6G7
P04050
P07711
Q92973
P0A7U3
P0A7V3
P36057
Q5TZY3
P06616
P07274
P07339
P0A7A9
P01857
P06709
P19367
P30153
P32602
P14294
P0AE18
P17169
P69222
Q15370
P0ABZ6
P29466
Q16773
P01040
P52565
P20618
P00374
P20434
P35244
P42330
P15303
Q9UKK6
P14126
P63104
P35813
P0AFG8
P08373
P32449
P0AB71
P08559
P25451
P0ADZ4
O15392
P29460
P01009
gi:51013357
P09038
P04951
P29972
P0A858
P10507
P0A6X7
P30086
P38197
Q5T937
P31373
Q13867
P12956
P29459
P62495
Q16658

P23721
P00579
P08879
P16435
P0AGD7
P21549
P14555
Q6NR85
P0AG44
P62166
P61316
P0A7D1
P0A7C2
Q549E4
P07256
P23909
Q09161
Q06124
Q12522
P07342
Q24117
P46680
P05100
Q9NWT6
P06132
Q5T8W2
P0AE67
P07012
P06756
P61765
P25098
O75531
P00128
P0AG86
P04451
Q5R1M2
P29547
P0A7Y4
O75469
P00560
P22213
P02766
P0A796
P0A817
P0A855
P61175
P68135
P0A7X3
P49888
P0A890
P05091
P08518
